# Factors affecting self-regulated learning in medical students: a qualitative study

**DOI:** 10.3402/meo.v20.28694

**Published:** 2015-11-06

**Authors:** Zahra Jouhari, Fariba Haghani, Tahereh Changiz

**Affiliations:** Department of Medical Education, Medical Education Research Center, Isfahan University of Medical Sciences, Isfahan, Iran

**Keywords:** medical student, self-regulation, learning, qualitative research

## Abstract

**Introduction:**

Clinical courses are required of all medical students and means that they must develop the key skill of self-regulation during learning. The ability to self-regulate learning strategies is affected by different factors. This study determined the views of medical students on the factors affecting self-regulated learning (SRL).

**Method:**

This study uses a qualitative approach and the content analysis method. Nineteen medical students in their fourth, fifth, and sixth years of study at Isfahan University of Medical Science participated in semi-structured, in-depth interviews. The students were selected using purposive sampling based on their overall grade point average (GPA).

**Results:**

Five main themes were found to affect SRL. These themes included family with the two subthemes of family supervisory and supportive roles; peers with the two subthemes of facilitating and inhibiting roles; instructors with the two subthemes of personal and educational instructor's characteristics; educational environment with the two subthemes of facilitator and inhibitor roles; and student with the two subthemes of facilitating and inhibiting personal factors.

**Conclusion:**

The outcomes of student understanding of the factors affecting self-regulation indicate that facilitating factors should be used on an individual basis to reduce the effect of inhibiting factors to improve self-regulation in students.

A recent concept in contemporary education is self-regulated learning (SRL), which is now considered to be a pillar of education (1). Educational self-regulation is of interest to educators and psychologists. Researchers now apply self-regulatory principles to academic study and other forms of learning, such as social and motor skills (2, 3).

The concept of self-regulation was first introduced in the social cognitive theory of Bandura (4). The main assumption of this theory is that each person is the outcome of personal, environmental, and behavioral variables (5, 6). Zimmerman and Pons (7) defined SRL strategies as learning which the learners personally initiate and conduct instead of relying on educators, parents, or other educational factors. Pintrich (8) defines SRL as an active, constructive process by which the learners set goals, monitor their learning, and control their motivation, behavior, and cognition.

Self-regulation of motivation involves controlling motivational beliefs such as self-efficacy, goal orientation, and task value. Self-regulation of behavior involves active control or use of resources available to students such as time, study environment, effort, and peers. Self-regulation of cognition involves the use of cognitive strategies such as rehearsal, organization, elaboration, and critical thinking (8). The main principle of SRL is that students who personally regulate their learning process will learn more effectively (9).

Kitsantas et al. (10) showed that SRL improves academic results. Dunn et al. (11) studied pathophysiology students and found that, despite the difficulty of the course, the use of SRL strategies had positive results for both students and instructors. SRL is based on active participation of students in personal, behavioral, motivational, and cognitive efforts to achieve valuable academic goals (12). Empirical studies have shown a significant relationship between academic success, use of self-regulation skills, and understanding ways of practicing self-regulation (13, 14).

Medical students must use key self-regulating skills in clinical courses that involve specialized aspects such as dealing with patients, meaningful learning, use of critical thinking, self-assessment of activities, and need for updating personal information (15). A study of college students showed that effective teaching methods such as problem-based learning (PBL) provide opportunities for participation in SRL (15). Randi (16) found that self-regulation by teachers in the classroom can cause learners to self-regulate. Self-regulation as a dependent variable can be influenced by gender, educational environment, teaching methods, personality, family background, and other variables (17). This means that self-regulation will be different in different contexts (18).

Students participating in clinical courses have a background in academic studies and are experiencing the last stages of official academic education in which they can learn the skills necessary to be lifelong learners (19). Sandars (20) stated that integration of self-regulation into the medical curriculum makes it possible to improve the clinical skills of medical students. Also, he considers most theoretical self-regulation to be applicable to medical education (20). The varieties of student activities during this period, especially at the bedside, provide different circumstances for training (11).

Research on medical students showed that adopting self-regulation of effort, time, and study environment can positively influence academic achievements (21). Self-regulated learners assess their performance, which then forms the basis for other efforts to regulate motivation, behavior, and context (22). West and Sadoski (23) confirmed that study strategies, especially self-testing, are strong predictors of medical student grades in the first semester of medical school.

There are few studies in Iran on SRL before and after academic education and the factors affecting self-regulation in medical students remain ambiguous. Research is needed on the development of self-regulatory processes and factors that affect SRL. This type of research will increase understanding of when students are at risk and factors that may promote motivation and self-regulation (8). Such knowledge is useful when designing curricula and classrooms that allow for self-regulation, and can suggest ways of teaching students to modify their strategies to fit the context (24) Because quantitative studies cannot provide answers to these issues, the present qualitative study sought the views of medical students on factors affecting SRL.

## Materials and methods

A qualitative content analysis approach was employed to gain better and deeper understanding of the viewpoints of students on factors affecting self-regulation of learning.

### Participants

The study population consisted of medical students (560 students) at Isfahan University of Medical Science in their fourth to sixth years of education during the 2014 academic year. Purposive sampling was employed for selection of students with the best knowledge about study topics (25). The students were selected because they had the best and most complete information about the topic of self-regulation in learning, its inhibitor, and facilitator factors. The researchers determined classrooms and hospitals for the sampling environments.

After receiving authorization from the educational deputy of the university, the first, second, and third authors (ZJ, FH, and TC, respectively) acquired a list of all fourth-, fifth-, and sixth-year students sorted by grade point average (GPA) from the Department of Medicine. To assure maximum variation in the samples, students with GPA considered to be high (>16), average (14–16), and low GPA (<14) were included in the study. GPA was used for sampling because research on learning and self-regulated study strategies highlighted the relationship between self-regulation and GPA (26). The studies have shown that there were important differences between the different self-regulation skills of university students with high GPA and those with low GPA (27, 28). Schutz et al. (29) divided a sample of trimester-one chiropractic students into high- and low-GPA groups and found that the high-GPA group scored significantly higher on study strategies.

In qualitative studies, data saturation indicates when interviews are complete (30); therefore, sample size cannot be predicted and is determined during the study. In this study, sampling was continued until data saturation. When data became repetitive and new information was no longer obtained from new interviews, sampling was considered complete (31). Data saturation occurred after 21 interviews with 19 students. Two students with more experience and information were interviewed twice in two sessions.

### Data collection

Semi-structured, in-depth interviews were conducted with each participant to gather the necessary information. The first students were invited to participate over the phone by the first author (ZJ). Before each face-to-face interview, ZJ explained the goals of the study to the participants and asked them to allow the interviewer to record the interview on a digital recorder. Participants were assured of the confidentiality of their personal information and reminded that participation in the study was voluntary. The major question ‘What factors affect your self-regulation in learning?’ was introduced as the interview guide. Probing questions focused on facilitating and inhibiting factors of SRL.

The first interview involved training of ZJ by the second author (FH), who has substantial experience in qualitative studies. All interviews were conducted in the Office of Educational Development at Isfahan University of Medical Science by ZJ in a room prepared for the convenience and comfort of the participants. The length of each interview varied from 25 to 90 min and was based on the experience and the amount of information that the participant presented. Notes were taken during the interviews by the interviewer (ZJ). These notes were used to verify the extracted codes. At the end of each interview, a gift was presented to the participants as a way of thanking them for their participation. All interviews were concluded within a 3-month time period.

### Data analysis

Conventional content analysis was employed to analyze the information gathered; this is a suitable method for extracting reliable and credible results from text data (32). This method allows creation of new knowledge and ideas, presentation of facts, and compression of extensive descriptions of a phenomenon, and results in concepts and descriptive categories (32). The information was organized using MAXQDA software (version 10, package series) (33).

Based on recommendations by Graneheim and Lundman (34), for the content analysis, meaning units were specified, and then the related codes were extracted and categorized based on similarity. When there was a high degree of abstraction, the themes were determined (34). By doing this, all interviews were immediately transcribed word-by-word by ZJ. The names of the participants were replaced with numerical codes. The messages inherent in each bit of text, phrases, and sentences were selected as meaning units. Then, the meaningful phrases, sentences, and words were determined and identified using initial codes. ZJ and FH independently read the first three transcripts and assigned the initial codes. Emerging differences were discussed and resulted in slight alterations to the coding structure. ZJ then coded the remaining transcripts. The similarities and differences between initial codes were examined and categorization was carried out by all authors (32). The aim was to reduce the number of categories by integrating some that were similar into broader categories. The categories were labeled definitively and the main themes were grouped by ZJ, FH, and TC.

In qualitative research, the concepts of credibility, confirm ability, dependability, and transferability have been used to describe aspects of trustworthiness (31, 35). Research credibility refers to confidence in how well the data and processes of analysis address the intended focus (34). Lincoln and Guba (35) referred to credibility as the ‘believability’ of a study. They intended credibility to be analogous to internal validity in quantitative research (35). To assure the credibility of the data, a researcher must be immersed in it; therefore, each interview was played several times and the transcript was reviewed by ZJ and FH. We tried to increase the credibility of the data by keeping prolonged engagement in the process of data collection and analysis.

Confirm ability refers to the degree to which findings of a study are genuine reflections of the participants investigated (35). To assure similarity between the codes determined and the experiences of the participants, the member check method was employed. The data and codes obtained from the interviews were presented to the participants for review, correction, and confirmation (30, 36). The initial codes and main themes were also evaluated by the peer-debriefing method. Consulting with a peer, or peer debriefing, allows for another check outside of a designated research team. Peers can be interested colleagues, classmates, or individuals within the community or within the phenomenon investigated (37). Peers should play the devil's advocate in that, while they are supportive of the clinician or educator's research efforts, they also serve as another vehicle to challenge the finding (38). ZJ and FH compared their results and discussed their differences and, if necessary, referred back to the text of the interviews (30, 36). The researchers worked until they reached a consensus on the main themes.

Another aspect of trustworthiness is dependability. Lincoln and Guba (35) stated that dependability refers to the consistency of study results over time and across researchers. This is similar to the concept of reliability in quantitative research (38). To provide dependability, all participants were interviewed in the same location and researchers used similar methods to code the data. The audit trail provides physical evidence of systematic data collection and analysis procedures for an external check. An expert researcher in qualitative studies who was a PhD student in medical education, native Iranian, and had no influence on the authors or the institution conducting the study reviewed physical evidence. The codes and themes were presented to two experts in qualitative studies for addition external cross-checking who were native Iranian and had expertise in self-regulation and medical education in general.

It is valuable to provide a clear and distinct description of the culture and context, selection and characteristics of participants, data collection, and process of analysis to facilitate transferability. A rich and vigorous presentation of findings together with appropriate reference to quotations from the interviews also enhances transferability (34). Transferability is similar to external validity in quantitative research (35). This study used maximum variation in sampling, described the context, data collection, participant characteristics, and process of study in detail to increase transferability of information (39). The study was approved by the Research Ethics Committee at the College of Medicine Research Center at Isfahan University of Medical Science.

## Results

A total of 21 interviews were conducted with 19 students (two students were interviewed twice). The participants comprised 10 males (52.3%) and nine females (47.7%). The average age of the participants was 22.79±1.58 years and the average GPA was 15.27±2.23 years. After categorization and integration of results, five main themes were obtained (Table 1).

**Table 1:** Themes and subtheme from interview with medical students

Theme	Subtheme
1. Family	a. Supportive
	b. Supervisory
2. Peers	a. Positive
	b. Inhibitor
3. Instructor's characteristics	a. Personal
	b. Educational
4. Educational environment	a. Facilitator
	b. Inhibitor
5. Student (self)	a. Personal facilitating factors
	b. Personal inhibiting factors

### Family

It was the view of the participants that family can play a supportive and supervisory role in self-regulation.

#### Supportive

The family environment, emotional and psychological support, and the experiences of other family members are components that can affect the self-regulation of students.

Participant 6: When I see that everyone in the family tries to create a calm environment and even sacrifices their own schedules to support my studies, this is certainly a great help in implementing my study schedule.

Participant 11: I think the family role is very important. If they have experienced academic education, they can be a great help. There are several people in my family with higher degrees who help me a great deal.

#### Supervisory

Students considered keeping track of academic achievements to be part of the supervisory role of the family and this supervision improved their concentration and focus on their academic activities.

Participant 12: At first, my family greatly controlled my education, but now they ask more general questions. My father is a physician and he is familiar with my coursework. He often asks me about the courses I'm taking and helps me to pay more attention to my studies.

### Peers

Students expressed both facilitating and inhibiting influences from peers on self-regulation.

#### Positive

Participants pointed to the educational and motivational role of peers; they learned from experiences of peers and received motivation from peers as positive aspects for SRL.

Participant 10: My classmates are very important to me. They can help me to study and have a positive influence on my studies.

Participant 4: Seeing one of your classmates doing something motivates you to do it as well. For example, if I'm being lazy, they can remind me and ask me to do the work with them.

#### Inhibitor

Participants pointed to inhibiting influences on self-regulation by peers as being a negative outlook about the educational environment, lack of motivation, and lack of success.

Participant 15: If I'm in a group where no one wants to do anything, it's not good. When someone does better than you, you try to plan and study more in order to succeed.

Participant 4: When I was at the university hospital, no one was motivated. They arrived late in the morning and were unenthusiastic even when the professor was present. This lowered my motivation and I think I studied less when I was there.

### Instructors

Students believed that instructors can greatly affect their self-regulation. The subcategories were the personal and educational characteristics of the instructors.

#### Instructor's personal characteristics

The experiences of the participants showed that a motivated and responsible instructor can improve self-regulation in the students. Communication and interaction between instructors and students, the role model presented by the instructor, and the seriousness of the instructor in following up on student activities can also affect self-regulation of students.

Participant 11: Your instructor in the hospital is very effective. We had instructors that lectured us about both study planning and ethics. Their instructions were perfect and effective.

Participant 4: If a student sees that his instructor teaches something one day and remembers to ask about it the next day and points out the mistakes, it can have a very positive effect.

Participant 9: Instructors, the educational environment, and assignments have a great influence on our self-regulation. For example, a suitable relationship between instructor and student can have a positive effect on student education.

#### Instructor's educational characteristics

The expertise's instructors, their lesson-planning, teaching methods, provision of timely and suitable feedback to students, motivation of students, and engagement with students in class discussions are educational characteristics of instructors that can have positive effects on student self-regulation.

Participant 17: The instructor presentation can affect our motivation. Sometimes you don't like a subject, but the instructor presents it so well and you like studying it. There are subjects in medicine that you don't like, but have to study. If they present them well, the student becomes motivated to study them.

Participant 13: The instructor teaching method helps students. For example, instructor A presents all his classes energetically and effectively, doesn't waste time without teaching you something, gives you assignments and asks questions of everyone, and then gives you a quiz. This is very effective.

### Educational environment

The experiences of the students showed that the atmosphere and conditions of the educational environment, educational planning, support services, and educational content can work as facilitating or inhibiting factors for self-regulation of the students.

#### Facilitator

Participation of students in educational programs, an appealing environment and suitable equipment, support services such as advisers and related educational courses, and applicability of the curriculum can facilitate self-regulation in the students.

Participant 13: One of the most important factors of self-regulation in the university is have a suitable environment and suitable equipment. For example, the availability of suitable equipment can even motivate students that don't like to study, like having a new medical moulage instead of working with an old or broken one.

Participant 5: University administrators must pay attention to the education program because it's very effective. A good educational program can motivate students and guide them to studying with motivation and interest, but an unsuitable program can work in reverse.

#### Inhibitor

Factors such as confusion in new students caused by planning mistakes, mandatory lesson systems, score-based education, and the length of medical education can work as inhibiting factors in the educational environment for self-regulation by students. The large volume of curriculum, lack of relationship between some subjects and future career, delayed access to education results, and stress-inducing medical courses can inhibit self-regulation in students.

Participant 16: The basic science courses have become too theoretical and boring. After a time, students will feel like the university is like high school. For three years we studied about parasites and fungi and … you won't use this at all later.

Participant 3: There are things we have to study that might be useful in the future, but right now they are not useful.

### Student (self)

Participants considered the personal characteristics of the students to be a factor affecting self-regulation.

#### Personal facilitating factors

Factors such as having initial knowledge about self-regulation, being motivated, having high self-esteem and self-efficacy, a successful academic record, personal desire, positive attitude, confidence, self-encouragement, and religious beliefs were factors facilitating self-regulation in students.

Participant 8: I think the most important factor for facilitating self-regulation is feeling that your actions affect your success; this can motivate you.

Participant 7: Your own experience at different stages of life greatly affects you. You need to plan for your work; we saw how planning produces results in high school exams and at university. These experiences are important.

Participant 7: Optimism is related to self-esteem. People with high self-esteem are more optimistic and can better regulate their schedules.

#### Personal inhibiting factors

Students stated that inability to use self-regulation strategies, such as inability to make daily schedules, lack of a defined goal, lack of concentration, and personal factors such as stress, pessimism, lack of motivation, carelessness, and lack of interest in the field of study can inhibit SRL. Other personal inhibiting factors include preoccupation with areas not related to education, such as worrying about future job prospectives, prioritizing non-academic activities, and financial problems.

Participant 15: It is necessary for student success that they don't become pessimistic and that they have a reachable goal that, if you try hard enough, you will attain. If one becomes pessimistic, he will stop working and trying.

Participant 16: The person himself is very effective. Ninety percent of self-regulation depends on regulating his/her schedule. If someone isn't motivated, even if you force them to study, they will give up after a few days.

Participant 3: I have a friend who knows he must study, but he prefers other work to this study. His first priority isn't studying.

## Discussion

The factors affecting SRL that were identified in this study included family, peers, instructors, educational environment, and personal characteristics of students. One of the most important focuses for instructors in educational institutions is to understand the factors that lead to better success for their pupils (40). Identifying such factors can lead educators toward better educational methods. Strage (17) concluded that a positive attitude by parents toward self-regulation and their support of students can facilitate SRL during academic endeavors. The present study found that students believed that the family environment and family emotional support facilitated self-regulation in students.

Students stated that peers can have a positive effect on SRL by providing motivation and educational support to their friends and by sharing their experiences. Woods reported that educational models at this stage of education provide suitable opportunities for participation in SRL. A careful study of students showed how they use self-regulation to better understand their study subjects. Woods (15) also conducted focused discussion sections with students in a surgery course and asked them to share experiences, strategies and behaviors that could help them to better understand their subjects.

The opinion of the participants in the present study was that the personal and educational characteristics of instructors were important for guiding students toward self-regulation. Several other studies confirm these results. Al Kadri (19) investigated the effects of clinical supervision and assessment and strategic features of study methods of medical students. He conducted a phenomenological study of different student groups and interviewed clinical instructors. He found that assessors and clinical instructors can affect the stress levels of their students (19). The results of a qualitative study by Endedijk (41) showed that self-regulation by instructors in defining the initial learning goals can practically affect self-regulation of their pupils. Educator conduct during class and their own self-regulation can encourage self-regulation in their students (16, 42). The use of effective teaching methods can also improve self-regulation in students (43). The results of a study on nursing students showed that PBL improves self-regulation among students (44).

The participants in the current study believed the environment of the university affected self-regulation. They stated that an appealing educational environment with applicable curriculum that encourages the student participation can facilitate self-regulation by students. Zimmerman and Risemberg found that the cognitive dimensions of self-regulation include motivation, application strategies, self-awareness about results, and sensitivity toward environmental and social contexts (45). The results of a study on 304 medical students at different educational levels showed that the perception of the students about their environment can affect self-regulation of learning (46). The participants in present study stated that the volume of contents and the environment of clinical courses were stress inducing. The results of a survey of medical students using a questionnaire sent by email showed that busy schedules and stress-inducing environments of medical studies required that students use skills such as main idea selection, stress control, metacognitive skills, and other self-regulation activities (47). Boekaerts and Cascallar (48) investigated self-regulation in students and discovered that the environment and atmosphere of classes can directly affect the motivation and use of self-regulation in students and that by understanding these effects, instructors can make better use of their environment. The results of a study from Iran showed that educational workshop in university improved study aids, time management, and self-exam skills in medical education (49).

The students interviewed in this study stated that motivation and self-efficacy can improve the use of self-regulation strategies in students. Stegers-Jager (50) conducted a retrospective study on 672 freshman medical students for 2 years. He investigated the relationship between student motivation and skills as affected by the use of learning strategies. The results showed that high motivation and self-efficacy can improve the abilities of medical students (50). Pizzimenti (51) showed that medical students with higher motivation during anatomy class showed better abilities.

The participants in this study believed that hopelessness, stress, anxiety, and lack of motivation are barriers to self-regulation in students. Van Nguyen (52) found that medical students with high levels of stress and anxiety made less use of self-regulation. Abdulghani (53) in a qualitative study on medical students showed that internal motivation is an important stimulus for use of self-regulation strategies and better academic performance. The results of a study in Pakistan showed a meaningful relation between the scores of students participating in online courses and self-efficacy, goal-setting, and learning control scores (54). The participant views were that personal desires and motivation are very important for use of self-regulation strategies. They stated that personal desires and internal motivation can affect other factors.

### Limitations of study

These study findings are not without potential limitation. First, the results of this study were limited to the viewpoints of students at a single university and students in the fourth, fifth and sixth years of study. Although the authors provided maximum variation in sampling and participant characteristics, results from other institutions or student populations may vary. Second, researchers found it difficult to coordinate with low-GPA students because they were mostly unwilling to participate in the study. Therefore, the researchers tried to increase confidence in participants for participation in the interviews. They promised participants free consultation after the interview. Third, only one interview was conducted with each student with the exception of two students who received two interviews. Researchers received detailed information with more interviews. In addition, receiving final approval of the transcribed interviews by email from the participants was at times a lengthy procedure and study time increased.

## Conclusion

Self-regulation in learning remains a critical skill to develop in medical students. The findings of this study show the viewpoints of students about factors affecting self-regulation in learning. Some, such as lack of ability in use of self-regulation strategies, lack of self-efficacy, and stress, can be ameliorated by interaction with student counselors. Others, such as the large volume of contents, lack of relationship between contents and future career, and cooperative learning, can be encouraged by a change in curriculum. It is suggested to conduct similar studies on a wider scale with a more variable population of students to better understand the factors affecting self-regulation in students.
